# *TREML4* mRNA Expression and Polymorphisms in Blood Leukocytes are Associated with Atherosclerotic Lesion Extension in Coronary Artery Disease

**DOI:** 10.1038/s41598-019-43745-y

**Published:** 2019-05-10

**Authors:** Victor Hugo Rezende Duarte, Carolinne Thaisa de Oliveira Fernandes Miranda, Marina Sampaio Cruz, Jéssica Nayara Góes de Araújo, Mychelle Kytchia Rodrigues Nunes Duarte, Ayda Maria Quirino Silva dos Santos, Isabelle Cristina Clemente dos Santos, Jéssica Cavalcante dos Santos, Ananília Medeiros Gomes da Silva, Juliana Marinho de Oliveira, Maria Sanali Moura de Oliveira Paiva, Marcos Felipe de Oliveira Galvão, Adriana Augusto Rezende, Mario Hiroyuki Hirata, Rosario Dominguez Crespo Hirata, André Ducati Luchessi, Vivian Nogueira Silbiger

**Affiliations:** 1 Department of Clinical and Toxicological Analysis, Federal University of Rio Grande do Norte, Natal, RN Brazil; 2 Department of Cardiology, Hospital Universitário Onofre Lopes, Natal, RN Brazil; 3 Faculty of Pharmaceutical Sciences, University of São Paulo, São Paulo, SP Brazil

**Keywords:** Calcification, Transcriptomics, Transcriptomics, Myocardial infarction, Diagnostic markers

## Abstract

Members of the triggering receptor expressed on myeloid cells (TREM) family are associated with atherosclerosis risk and progression. *TREML4* is upregulated in the early phase of acute coronary syndrome. We investigated the relationship between the mRNA expression of 13 genes in blood leukocytes, *TREML4* polymorphisms, and coronary artery lesion extension (Friesinger index) in patients with coronary artery disease (CAD) (n = 137). *TREML4* rs2803495 (A > G) and rs2803496 (T > C) variants and leukocyte mRNA expression were analysed by qRT-PCR. *TREML4* expression was higher in patients with major coronary artery lesions than in subjects without or with low and intermediate lesions (*p* < 0.05). However, *TREML4* polymorphisms were not associated with coronary lesion extent. Presence of the rs2803495 G allele was not associated with increased *TREML4* mRNA expression. Patients carrying the rs2803496 C allele (TC/CC genotypes) were more likely to express *TREML4* mRNA than non-C allele carriers (allele C: OR 7.3, and 95% CI 1.9–27.5, *p* = 0.03). In conclusion, increased *TREML4* mRNA expression in blood leukocytes is influenced by gene polymorphisms and is associated with more severe coronary artery lesions, suggesting its potential as a biomarker of the extent of coronary lesions in patients with CAD.

## Introduction

Cardiovascular disease (CVD) remains among the leading causes of mortality and morbidity in developed and developing countries^1^. Therefore, a better understanding of the disease aetiology and more efficient therapies are needed. Genetic predisposition as well as environmental factors and lifestyle are thought to contribute to disease risk^2^. Cardiovascular risk is assessed mainly on the basis of classic risk factors, such as age, gender, smoking status, blood pressure, glucose levels, and lipid levels. However, studies have shown that at least 15% of patients with coronary artery disease (CAD) do not present classic risk factors, and some have poor prognosis^3,4^.

In the last decade, 164 genetic loci conferring a modest risk for CAD have been identified^5^. Genome-wide association studies investigating mechanistic links between disease onset and genetic variation aimed at identifying novel treatment targets have been conducted. For example, *PCSK9*, *GUCY1A3*, *ANGPTL4*, and *ANGPTL3* are associated with CAD and are potential druggable targets^6^. The discovery of novel circulating biomarkers involved in atherosclerosis pathophysiology may improve cardiovascular risk assessment^7^.

Blood is a source of transcriptomic CVD-related biomarkers. In a microarray-based study, we observed that the mRNA expression of 13 genes (*ALOX15*, *AREG*, *BCL2A1*, *BCL2L1*, *CA1*, *COX7B*, *ECHDC3*, *IL18R1*, *IRS2*, *KCNE1*, *MMP9*, *MYL4*, and *TREML4*) in blood leukocytes was increased within 2 h after the initial episode of acute coronary syndrome (ACS). Therefore, these genes were suggested as potential expression biomarkers for very early stages of ACS^8^. An integrative follow-up study using transcriptomics, genomics, proteomics, and next-generation sequencing strategies revealed that the mRNA expression of *TREML4* in leukocytes and two *TREML4* polymorphisms (rs2803495 and rs2803496) are associated with a high degree of coronary artery calcification (CAC) in patients with CAD^9^. Triggering receptor expressed on myeloid cells-like protein 4 (TREML4) is a TREM family receptor that is highly expressed in CD8α+ dendritic cells and splenic macrophages. This protein is involved in the capture, processing, and presentation of antigens by major histocompatibility complex class I and class II proteins^10,11^.

Based on our previous research, TREML4 likely is involved in the atherosclerotic process and may have potential as a biomarker of cardiovascular risk. However, few studies have investigated its relationship with CVD and more research is necessary to determine the role of TREML4 in the atherosclerotic process and coronary lesion development. Therefore, this study aimed to evaluate mRNA expression of the above 13 genes in peripheral blood leukocytes of patients with suspected CAD undergoing coronary angiography and to assess the association between *TREML4* mRNA expression and polymorphisms as a potential biomarker for investigating the extent of coronary lesions.

## Results

### Clinical and biochemical laboratory data

Atherosclerotic lesion/CAD was detected in 74.45% of subjects (n = 102). Assessment of the extent of coronary artery lesion revealed that 38 (27.7%) patients with CAD had low lesions (FI 1–5), 41 (29.9%) had intermediate lesions (FI 6–10), and 23 (16.8%) had major lesions (FI 11–15). Thirty-five (25.5%) subjects had no atherosclerotic lesions and were classified as the control group (FI = 0). Clinical and biochemical laboratory data are shown in Table 1. The mean age was higher in patients with intermediate and major artery lesions than in the control group (*p* = 0.002 and 0.001, respectively). Patients with intermediate lesions had higher systolic pressure than control subjects (*p* = 0.01) and low-lesion patients had higher BMIs than those without lesions (*p* = 0.02). Moreover, 60% of patients with major artery lesions frequently used statins, which was significantly higher than the use in the other groups (*p* = 0.002).

**Table 1:** Clinical and laboratory data of patients classified according to coronary artery lesion extension.

Variables	Without (35)	Extension of coronary lesion		Major (23)	*p*
		Low (38)	Intermediate (41)		
Age, years	53 ± 10^a,b,c^	60 ± 10	61 ± 9	63 ± 7	<*0.001*
Sex male, %	45.7 (16)	65.8 (25)	56.2 (23)	43.5 (10)	*0.242*
BMI, kg/m^2^	29.2 ± 5.0	25.7 ± 6.1	27.1 ± 4.7	27.7 ± 4.6	*0.043*
Obesity, %	37.1 (13)	10.8 (4)	19.6 (8)	14.3 (3)	*0.155*
Dyslipidaemia, %	82.9 (29)	84.2 (32)	90.0 (36)	78.3 (18)	*0.640*
Diabetes, %	22.9 (8)	21.1 (8)	31.7 (13)	39.1 (9)	*0.380*
Hypertension, %	74.3 (26)	84.2 (32)	85.4 (35)	87.0 (20)	*0.520*
Diastolic pressure, mmHg	80 ± 10	84 ± 18	88 ± 18	82 ± 20	*0.115*
Systolic pressure, mmHg	134 ± 23^d^	141 ± 21	153 ± 37^d^	141 ± 22	*0.198*
Physical activity, %	31.4 (11)	44.7 (16)	39.0 (16)	31.8 (7)	*0.627*
Alcohol intake, %	32.4 (11)	39.5 (15)	17.1 (7)	34.8 (8)	*0.157*
Smoking, %	14.3 (5)	26.3 (11)	26.8 (6)	26.1 (6)	*0.539*
Family history of CAD, %	45.7 (16)	44.7 (17)	51.2 (21)	42.5 (10)	*0.918*
Glucose, mg/dl	104.6 ± 34.7	101.4 ± 29.0	134.0 ± 80.0	119.8 ± 47.7	*0.070*
Total cholesterol, mg/dL	174.6 ± 36.6	179.3 ± 55.7	183.7 ± 52.2	183.0 ± 54.1	*0.871*
HDL cholesterol, mg/dL	38.0 ± 12.8	35.6 ± 11.3	34.9 ± 10.1	40.3 ± 12.1	*0.262*
LDL cholesterol, mg/dL	101.4 ± 33.9	105.4 ± 47.5	113.1 ± 43.6	113.3 ± 44.1	*0.594*
Triglycerides, mg/dL	176.3 ± 99.4	191.9 ± 121.7	182.6 ± 130.2	146.8 ± 76.3	*0.496*
AST, U/L	36.6 ± 20.1	31.6 ± 13.9	31.0 ± 15.8	31.4 ± 18.4	*0.541*
ALT, U/L	31.2 ± 20.3	34.1 ± 15.4	27.9 ± 20.2	26.6 ± 16.8	*0.048*
Urea, mg/dL	35.8 ± 10.2	37.8 ± 9.4	38.3 ± 10.9	43.0 ± 17.2	*0.147*
Creatinine, mg/dL	0.87 ± 0.25	0.96 ± 0.25	0.90 ± 0.21	0.87 ± 0.33	*0.476*
Uric acid, mg/dL	5.0 ± 1.7	4.9 ± 1.6	4.4 ± 1.6	4.7 ± 1.3	*0.397*
Antihypertensive, %	62.9 (22)	78.9 (30)	65.9 (27)	73.9 (17)	*0.423*
Anticoagulants, %	25.7 (9)	31.6 (12)	41.5 (17)	30.4 (7)	*0.517*
Antidiabetics, %	20.0 (7)	13.2 (7)	17.1 (7)	34.8 (8)	*0.211*
Statins, %	14.3 (5)	26.3 (10)	29.3 (12)	60.9 (14)^d,e,f^	*0.002*

Data are shown as the mean ± standard deviation or the percentage for categorical variables (number of patients). Parametric analysis was performed by ANOVA. For non-parametric testing, the Kruskal–Wallis test was used. Categorical variables were compared by Chi-square test. BMI, body mass index; HDL, high-density lipoprotein; LDL, low-density lipoprotein; AST, aspartate aminotransferase; ALT, alanine transaminase. ‘Without’, Friesinger index 0; ‘Low’, Friesinger index 1–5; ‘Intermediate’, Friesinger index 6–10; ‘Major’, Friesinger index 11–15.

^a^*p* = 0.023, without vs low extension of coronary lesion by post-hoc Tukey test.

^b^*p* = 0.002, without vs intermediate extension of coronary lesion by post-hoc Tukey test.

^c^*p* = 0.01, without vs major extension of coronary lesion by post-hoc Tukey test.

^d^*p* < 0.010, without vs major extension of coronary lesion by Chi-square test.

^e^*p* = 0.007, low vs major extension of coronary lesion by Chi-square test.

^f^*p* < 0.013, intermediate vs major extension of coronary lesion by Chi-square test.

### mRNA expression and *TREML4* polymorphisms

Of the 13 genes analysed in this study, expression of only *TREML4* and *ECHDC3* was associated with the extent of coronary artery lesions (Supplementary Table 1). Forty-four percent of all patients tested positive for *TREML4* and *ACTB* expression in quantitative reverse transcription (qRT)-polymerase chain reaction (PCR), whereas 55.6% tested positive for *ACTB* alone.

**Figure 1:**
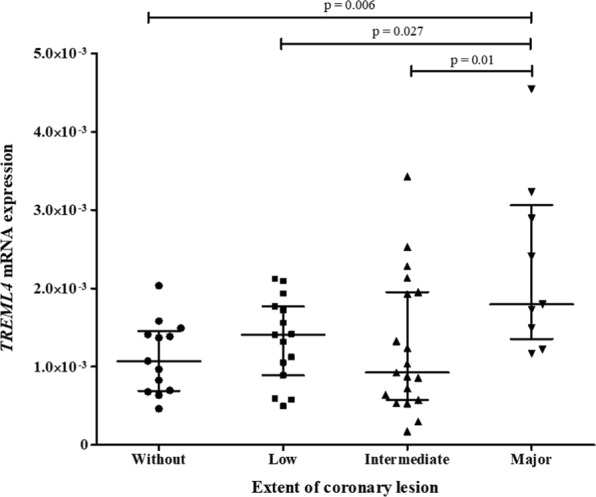
Relation between coronary artery lesion extension and TREML4 mRNA expression in leukocytes of CAD patients. Data are shown as the median and interquartile range and were compared using Kruskal–Wallis and Mann–Whitney tests. Relative expression was calculated using the 2^−ΔCT^ method, with *ACTB* as a reference gene. Number of samples in each group: without (13), low (15), intermediate (20), and major (9) artery lesions. ‘Without’, Friesinger index 0; ‘Low’, Friesinger index 1–5; ‘Intermediate’, Friesinger index 6–10; ‘Major’, Friesinger index 11–15.

The genotype frequencies of *TREML4* polymorphisms (rs2803495 and rs2803496) were in Hardy–Weinberg equilibrium, indicating that there were no genotyping errors inbreeding, and evolutionary pressure. The genotype and allele frequencies were similar among coronary artery lesions of different extents (Table 2). Linkage disequilibrium between rs2803495 and rs2803496 was not detected (D′ = 0.59). Moreover, subjects with the C allele were more likely to have high *TREML4* mRNA expression (OR 7.3, 95% CI 1.9–27.5, *p* = 0.03), as shown in Table 3. Patients with major artery lesions had 1.4-fold, 1.2-fold, and 1.4-fold higher *TREML4* mRNA expression levels than those with intermediate (*p* = 0.01) and low (*p* = 0.027) artery lesions and controls (*p* = 0.006), respectively (Fig. 1).

**Table 2:** Relationships of *TREML4* polymorphisms with coronary artery lesion extension in CAD patients.

Polymorphism		All	Extension of coronary artery lesion				*p*
			Without	Low	Intermediate	Major	
rs2803495 A/G		N = 136	N = 33	N = 39	N = 40	N = 22	
Genotypes, %	AA	77.9 (106)	78.8 (26)	82.1 (32)	75.6 (31)	73.9 (17)	*0.813*
(Codominant)	AG	20.6 (28)	21.2 (7)	17.9 (7)	22.0 (9)	21.7 (5)	
	GG	1.5 (2)	0	0	2.4 (1)	4.3 (1)	
(Dominant)	AA	77.9 (106)	78.8 (26)	82.1 (32)	75.6 (31)	73.9 (17)	*0.863*
	AG + GG	22.1 (30)	21.2 (7)	17.9 (7)	24.4 (10)	26.1 (6)	
Alleles, %	A	88.2 (134)	89.4	91.0	86.6	84.8	*0.702*
	G	11.8 (2)	10.6	9.0	13.4	15.2	
**rs2803496 C/T**		**N** = **134**	**N** = **31**	**N** = **39**	**N** = **40**	**N** = **23**	
Genotypes, %	TT	76.9 (103)	83.9 (26)	71.8 (28)	80.5 (33)	69.6 (16)	*0.526*
(Codominant)	CT	22.4 (30)	16.1 (5)	28.2 (11)	17.1 (7)	30.4 (7)	
	CC	0.7 (1)	0	0	2.4 (1)	0	
(Dominant)	TT	76.9 (103)	83.9 (26)	71.8 (28)	80.5 (33)	69.6 (16)	*0.492*
	CT + CC	23.1 (31)	16.1 (5)	28.2 (11)	19.5 (8)	30.4 (7)	
Alleles, %	T	88.1 (133)	91.9	85.9	89.0	84.8	*0.620*
	C	11.9 (1)	8.1	14.1	11.0	15.2	

Numbers of subjects are indicated in parentheses. Frequencies were compared by the Chi-Square test. *p*-Values refer to comparisons among genotype frequencies of the different models and among allelic frequencies. For less than 10% of all samples, the genotype could not be determined. ‘Without’, Friesinger index 0; ‘Low’, Friesinger index 1–5; ‘Intermediate’, Friesinger index 6–10; ‘Major’, Friesinger index 11–15.

**Table 3:** Relationships of *TREML4* polymorphisms with blood leukocyte *TREML4* mRNA expression in CAD patients.

Polymorphism	Genotype	*TREML4 mRNA* expression		OR (95%CI)	*p*
		Low expression	High expression		
rs2803495 A/G	AA	66.7 (18)	76.9 (20)	1	*0.409*
	AG + GG	33.3 (9)	23.1 (6)	1.7	—
				(0.5–5.6)	
rs2803496 C/T	TT	84.6 (22)	44.0 (11)	1	*0.03*
	CT + CC	22.0 (4)	56.0 (14)	7.3	—
				(1.9–27.5)	

Univariate logistic regression analysis. *p*-Values refer to comparisons between genotype frequencies of the two polymorphisms. OR, odds ratio; CI, confidence interval; ‘Low expression’, *TREML4* mRNA expression bellow the median value; ‘High expression’, *TREML4* mRNA expression above median value.

Clinical and laboratory data of patients classified according to *TREML4* mRNA expression are shown in Supplementary Table 2. Diabetes mellitus was more frequent among patients who expressed *TREML4* mRNA above the median (*p* = 0.015), and these subjects more frequently used antidiabetics (*p* = 0.001). Interestingly, patients with diabetes mellitus using antidiabetic drugs had a greater likelihood of expressing *TREML4* mRNA at levels above the median (OR 3.630, 95% CI 1.238–10.644, *p* = 0.019 and OR 8.611, 95% CI 2.191–33.851, *p* = 0.002, respectively), as shown in Table 4.

**Table 4:** Relationships of diabetes mellitus, use of antidiabetics, and rs2803496 allele ***C*** with *TREML4* mRNA expression above the median value.

Model	Variables	Β	OR (IC 95%)	*p*
1	Diabetes mellitus	1.3	3.7 (1.1–11.8)	0.029
2	Antidiabetics	2.6	13.0 (2.2–33.9)	0.002
3	Diabetes patients	1.6	4.9 (1.2–19.1)	0.023
	rs2803496 C allele	2.2	8.9 (2.1–37.3)	0.003
4	Antidiabetics	3.0	20.9 (3.3–130.7)	0.001
	rs2803496 C allele	2.4	11.5 (2.4–54.8)	0.002

OR, odds ratio; CI, confidence interval. Dependent variable: *TREML4* mRNA expression below median value/above median value.

Model 1: Univariate logistic regression analysis. Independent variable: diabetes mellitus yes/no.

Model 2: Univariate logistic regression analysis. Independent variable: using antidiabetics yes/no.

Model 3: Multivariate logistic regression analysis, method forward stepwise. Independent variable: diabetes mellitus yes/no and rs2803496 allele C/T.

Model 4: Multivariate logistic regression analysis, method forward stepwise. Independent variable: using antidiabetics yes/no and rs2803496 allele C/T.

## Discussion

This study highlighted the association between *TREML4* mRNA expression and polymorphisms as a potential biomarker for coronary lesion extent. Chagas *et al*. studied 337 patients who were undergoing coronary angiography for suspected CAD and observed that anthropometric measures were not correlated with the extent of coronary lesions^12^. The authors found only a few associations between classic risk factors and atherosclerotic burden, similar to our findings shown in Table 1. Together, these observations strengthen the hypothesis that classic risk factors for CVD are not sufficiently sensitive to evaluate the extent of coronary lesions in patients with CAD, highlighting the importance of studies to discover novel non-invasive, specific, and sensitive biomarkers for early assessment of atherosclerotic lesions.

Although the 13 genes evaluated in this study reportedly are associated with ACS^8^, in this study, only two genes, *TREML4* and *ECHDC3*, were associated with coronary artery lesion extent. Hypotheses may explain these results are that *ALOX15*, *AREG*, *BCL2A1*, *BCL2L1*, *CA1*, *COX7B*, *IL18R1*, *IRS2*, *KCNE1*, *MMP9*, and *MYL4* are involved in atherosclerosis destabilization^7^. Importantly, the patients in the present study did not have destabilized atherosclerotic plaques. Therefore, we evaluated the physiological processes that precede ACS. Among the 13 genes associated with ACS, some reportedly are involved in atherothrombotic processes, e.g. *IRS2* and *MMP9* are involved in plaque disruption and acute myocardial infarction in humans^13–15^ and *KCNE1* is involved in ischemic events, such as acute myocardial infarction, in a rat model^16^.

Our finding that *ECHDC3* mRNA expression is associated with coronary lesion extent corroborates that of our previous study performed in a smaller population^17^. *TREML4* also has an important association with the extent of coronary lesion, but few studies have evaluated its relationship with the process of atherosclerosis. We previously observed increased *TREML4* expression in patients with ACS^8^. This result reinforces our findings, as ACS is a complex pathological process that predisposes for cardiac injury and a common risk factor in patients with CAD.

TREM and TREML are receptors composed of a single extracellular variable-type immunoglobulin-like domain and are structurally similar, with a transmembrane domain and a short cytoplasmic tail lacking any known signalling motif^18^. They are expressed predominantly on myeloid cells^19^. Studies have demonstrated that TREM clusters are involved in the development of atherosclerosis. Most studies on TREM clusters focused on two members, TREM-1 and TREM-2, both of which are involved in the innate immunity inflammatory process induced by Toll-like receptors. Considering that inflammation is an important mechanism in the atherosclerotic process, the association of *TREM-1* and *TREM-2* expression with atherosclerotic plaque formation^20,21^ and polymorphisms in *TREM-1* have been reported to be associated with CAD^20^.

In our study population, 22.1% of patients with CAD were carriers of the rs2803495 AG + GG genotypes and the minor-allele frequency of the G allele was 11.8% (Table 2). These results are similar to those in a European population in phase 3 of the 1000 Genomes project (http://www.internationalgenome.org/), in which 21.2% of subjects were carriers of the AG + GG genotypes and the G allele frequency was 13.0%. The SNP rs2803496 showed similar frequencies for CT + CC genotypes (23.1%) and C allele (11.9%), which are comparable to those found in the European population (16.2% and 11.0%, respectively). *TREML4* mRNA expression was higher in patients with CAD carrying the C allele (genotypes CT + CC) for rs2803496 in leukocytes than in non-C allele carriers (*p* = 0.030). In contrast, the G allele (genotypes AG + GG) for rs2803495 did not influence mRNA expression levels (*p* = 0.409). Similarly, Sen *et al*. reported that the presence of the C allele was strongly associated with increased *TREML4* expression in patients with CAC in the USA^9^. This is the first study to evaluate these polymorphisms and *TREML4* mRNA expression in CAD patients in an admixture Brazilian population.

Both rs2803495 and rs2803496 variants are in the 5′-untranslated region (UTR) of *TREML4*. The 5′-UTR contains regulatory regions, such as selenocysteine insertion sequence elements, AU-rich elements, riboswitches, and microRNA-binding sites that affect translational efficiency, subcellular localization, and mRNA stability^22^. Therefore, the 5′-UTR plays an important role in controlling gene expression^23^, and polymorphisms in this region may influence mRNA stability and, consequently, gene expression levels.

We previously reported a significant increase in *TREML4* mRNA expression in leukocytes of patients with ACS compared to in controls^8^. However, the role of this gene in the presence and progression of CVD remained unclear, and only two studies have evaluated *TREML4* in CVD. Studies on mRNA expression in the peripheral blood have revealed vascular inflammatory markers and other indicators of tissue injury reflecting cellular damage anywhere in the body^24^. In this study, *TREML4* mRNA expression was correlated with the extent of coronary lesions. Patients with major lesions showed higher *TREML4* expression than other groups (Fig. 1). Sen *et al*.^9^ evaluated leukocyte subpopulations of patients with CAC, and also detected higher *TREML4* expression levels by qRT-PCR in human leukocytes. CAC is well known to be closely related to atherosclerosis^25^. These results suggest that *TREML4* expression is involved in the progression and extent of coronary lesions, and its consequences, such as CAC. Therefore, *TREML4* expression is a potential marker of atherosclerosis severity and may be useful for monitoring patients with CAD.

A high frequency of diabetes mellitus (OR 3.7, CI 1.1–11.8, *p* = 0.029) as well as antidiabetic use (OR 13.0, CI 2.2–33.9, *p* = 0.002) were observed in patients who had high *TREML4* mRNA levels, and these patients also presented more extensive atherosclerotic lesions (Table 3). Hyperglycaemia, a diabetes mellitus symptom, is a well-established, important risk factor for CAD. Increased use of antidiabetics in the diabetes mellitus group was expected; however, these medications may be related to the progression of atherosclerotic lesions, as previous studies examining the use of some antidiabetics showed controversial results, in which mortality was not reduced^26,27^.

Our results suggest that *TREML4* mRNA expression in leukocytes is increased in patients with CAD with more extensive atherosclerotic lesions in the coronary arteries. Moreover, it is likely that carriers of rare alleles of *TREML4* polymorphisms are more prone to having more extensive coronary lesions. Both findings support that *TREML4* is involved in the formation, progression, and severity of atherosclerotic lesions. However, considering that the polymorphisms in this study are mutations in the 5′-UTR of mRNA, an important regulatory region for protein synthesis, further studies on the involvement miRNA, proteins, and *TREML4* in other CAD stages are necessary to confirm this hypothesis.

In conclusion, this study evaluated the relationship between gene expression and *TREML4* polymorphisms in patients undergoing coronary angiography. The results suggest that *TREML4* mRNA expression in leukocytes is influenced by the extent of coronary artery lesions and gene polymorphisms in patients with CAD. Therefore, the *TREML4* mRNA level may be an important biomarker for evaluating the progression and severity of CAD.

## Methods

### Study population

One hundred thirty-seven subjects aged 30–74 years undergoing coronary angiography for CAD diagnosis were enrolled in this cross-sectional study in our hospital, as previously reported^28,29^. Patients were selected at the Hemodynamics unit of the Hospital Universitário Onofre Lopes, Instituto do Coração, and Natal Hospital Center in Natal, Rio Grande do Norte, Brazil. This study was approved by the hospital’s Research Ethics Committee of the Hospital Universitário Onofre Lopes under protocol number 0001.0.051.294-11. Written informed consent was obtained from each participant prior to sample collection, and all experiments were performed in accordance with relevant guidelines and regulations.

Participants were asked to provide information about age, BMI, gender, hypertension, obesity, cigarette smoking status, physical activity, alcohol consumption, and family history of CAD. The following exclusion criteria were used: diagnosis of cardiomyopathy, heart valve disease, congenital diseases, pericarditis, chronic kidney disease, liver failure, endocrine disorder (except for type 2 diabetes), inflammatory diseases, malignant diseases, blood disorders, autoimmune diseases, family history of hypercholesterolemia, and previous cardiovascular events, such as ACS or coronary revascularization.

### Assessment of coronary lesion extent

The extent of coronary artery lesions was assessed using the Friesinger index (FI)^29^. Each of the three main coronary arteries (anterior descending, circumflex, and right coronary) was scored separately from zero to five. The scores were: 0, no arteriographic abnormalities; 1, trivial luminal narrowing <29%; 2, localized 30–68% luminal narrowing; 3, multiple 30–68% luminal narrowing; 4, 69–100% luminal narrowing without 100% occlusion of proximal segments; 5, total obstruction of a proximal segment. The FI value ranged from 0 to 15^29^. Based on the FI, subjects were categorized into four groups as described by Duarte *et al*.^17^ and Santos *et al*.^30^; ‘without lesions’, FI = 0, control group; ‘low lesion’, FI 1–5; ‘intermediate lesion’, FI 6–10; and ‘major lesion’, FI 11–15. All other patients with coronary artery lesion (FI ≥1) were considered as CAD diagnosis confirmed.

### Blood sampling and biochemical analysis

Peripheral blood samples were collected from patients before coronary angiography in tubes without anticoagulant for biochemical analysis and in tubes containing EDTA for DNA extraction. Blood leukocytes were isolated by centrifugation (1340 × *g*, 15 min) for RNA analysis. Fasting serum glucose, triglycerides, total cholesterol, high-density lipoprotein (HDL)-cholesterol, urea, creatinine, uric acid, alanine aminotransferase, and aspartate aminotransferase (AST) were measured using colorimetric and enzymatic colorimetric assays on a Labmax Plenno biochemical analyser (Labtest, Minas Gerais, Brazil). Levels of low-density lipoprotein (LDL)-cholesterol were calculated according to the Friedewald formula^31^.

### RNA isolation and mRNA expression analysis

Total RNA was extracted from leukocytes stored in RNAlater^®^ stabilization solution (Life Technologies, Carlsbad, CA, USA) using a RiboPure^TM^ Blood kit (Life Technologies). RNA integrity was assessed by 1% agarose (Ludwig Biotecnologia LTDA, Alvorada, RS, Brazil) gel electrophoresis in MOPS buffer. RNA concentration was measured using a Qubit RNA HS Assay Kit (Life Technologies) in a Qubit^®^ 2.0 Fluorometer (Life Technologies). RNA samples were stored at −80 °C. mRNA expression was analysed by qRT-PCR. cDNA was synthesised using a High-Capacity cDNA Reverse Transcription Kit (Applied Biosystems, Foster City, CA, USA) in a MyCycler Thermal Cycler (Bio-Rad, Hercules, CA, USA). qPCRs were run using TaqMan assays (*ALOX15*, Hs00609608_m1; *AREG*, Hs00155832_m1; *BCL2A1*, Hs00187845_m1; *BCL2L1*, Hs00169141_m1; *CA1*, Hs00266139_m1; *COX7B*, Hs00371307_m1; *ECHDC3*, Hs00226727_m1; *IL18R1*, Hs00175381_m1; *IRS2*, Hs00275843_s1; *KCNE1*, Hs00897540_s1; *MMP9*, Hs00957562_m1; *MYL4*, Hs00267321_m1; *TREML4*, Hs01080584_g1; Life Technologies). The reference gene was selected from a normalization study of three endogenous candidate genes: *GAPDH* (Hs.592355_g1), *ACTB* (Hs.520640_g1), and *18S rRNA* (Hs.626362_g1) using geNorm and NormFinder software. qPCRs were run in 96-well plates using the 7500 Fast Real-time PCR System (Applied Biosystems). Relative mRNA expression was calculated using the 2^–ΔCT^ method, with *ACTB* as a reference gene^32^.

### DNA isolation and genotyping

Genomic DNA was isolated from whole blood collected in EDTA tubes using the QIAamp DNA Blood Mini Kit (Qiagen, Hilden, Germany). DNA integrity was assessed by 0.8% agarose gel electrophoresis, and DNA concentration was measured using the Qubit dsDNA BR Assay Kit (Life Technologies) with the Qubit^®^ 2.0 Fluorometer (Life Technologies). DNA samples were stored at −20 °C until analysis. *TREML4* polymorphisms rs2803495 (A > G) and rs2803496 (C > T) were genotyped by qRT-PCR using TaqMan SNP Genotyping Assays (C_27302616_10 and C_27302614_10) (Life Technologies) in a 7500 Fast Real-Time PCR System (Applied Biosystems), according to the manufacturer’s protocol. Ten percent of randomly selected DNA samples were assayed in duplicate, and SNPs were 100% confirmed and concordant in the duplicate pairs.

### Statistical analysis

Statistical analysis was performed using SPSS^®^ 22.0 software (SPSS, Inc., Chicago, IL, USA). Normal distribution was evaluated using the Kolmogorov–Smirnov test. Continuous variables with normal distributions are presented as the mean and standard deviation and were compared using *t*-tests or analysis of variance followed by Tukey’s test. Variables without parametric distributions are presented as the median and were analysed using the Kruskal–Wallis test followed by the Mann–Whitney test. Categorical variables were compared by the chi-square test and Fisher exact test. Independent variables possibly affecting *TREML4* mRNA expression were determined by multivariate regression analysis. Genotyping analysis was performed using the *R* package v.3.3.1 (R DEVELOPMENT CORE TEAM, 2015). Chi-square analysis was utilized to test for Hardy–Weinberg equilibrium and to compare allele frequencies and genotype distributions. Logistic regression was performed using the *SNPassoc* package v.1.9–2. A *p*-value < 0.05 was considered significant. Linkage disequilibrium was evaluated using HAPLOVIEW^®^ 4.2 software^33^.

## Supplementary information

Supplementary Table 1, Supplementary Table 2.

## Data Availability

All data generated or analysed in this study are included in this article (and its Supplementary Information Files).
