# Supplementary material for: TREML4 mRNA Expression and Polymorphisms in Blood Leukocytes are Associated with Atherosclerotic Lesion Extension in Coronary Artery Disease
Source: Sci Rep. 2019 May 10;9:7229. doi: 10.1038/s41598-019-43745-y (PMC6510738; doi:10.1038/s41598-019-43745-y)
Supplement: Supplementary file 1 — Supplementary Table 1, Supplementary Table 2. [file 41598_2019_43745_MOESM1_ESM.docx]

***TREML4* mRNA Expression and Polymorphisms in Blood Leukocytes are Associated with Atherosclerotic Lesion Extension in Coronary Artery Disease**

Victor Hugo Rezende Duarte^1,#^, Carolinne Thaisa de Oliveira Fernandes Miranda^1,#^, Marina Sampaio Cruz^1^, Jéssica Nayara Góes de Araújo^1^, Mychelle Kytchia Rodrigues Nunes Duarte^1^, Ayda Maria Quirino Silva dos Santos^1^, Isabelle Cristina Clemente dos Santos^1^, Jéssica Cavalcante dos Santos^1^, Ananília Medeiros Gomes da Silva^1^, Juliana Marinho de Oliveira^2^, Maria Sanali Moura Oliveira Paiva^2^, Marcos Felipe de Oliveira Galvão^1^, Adriana Augusto Rezende^1^, Mario Hiroyuki Hirata^3^, Rosario Dominguez Crespo Hirata^3^, André Ducati Luchessi^1^ & Vivian Nogueira Silbiger^1,*^

^a^Department of Clinical and Toxicological Analysis, Federal University of Rio Grande do Norte, Natal, RN, Brazil

^b^Department of Cardiology, Hospital Universitário Onofre Lopes, Natal, RN, Brazil

^c^Faculty of Pharmaceutical Sciences, University of São Paulo, São Paulo, SP, Brazil

*Corresponding author

E-mail address: [viviansilbiger@hotmail.com](mailto:viviansilbiger@hotmail.com) (VNS)

Address: Department of Clinical and Toxicological Analysis, Federal University of RioGrande do Norte, Avenue General Gustavo Cordeiro de Farias, S/N, Natal, Rio Grande do Norte CEP: 59014-520, Brazil

^#^These authors contributed equally to this work and share first authorship.

| **Gene** | **Extension of coronary lesions** | | | | ***p*** |
| --- | --- | --- | --- | --- | --- |
|  | **Without (35)** | **Low (38)** | **Intermediate (41)** | **Major (23)** |  |
| *COX7B* | 0,0043796  (0,0019983–0,0142885) | 0,0042820  (0,0007483–0,0120235) | 0,0041548  (0,000944–0,0115898) | 0,0054579  (0,0018249–0,0206173) | 0.41 |
| *KCNE1* | 0,0000628  (0,0000041–0,0003174) | 0,0000682  (0,0000682–0,0002577) | 0,0000759  (0,0000077–0,0001775) | 0,0000567  (0,0000117–0,0001944) | 0.87 |
| *TREML4* | 0,0010738  (0,0004632–0,0020360) ^a^ | 0,0014081  (0,0004968–0,0021195) ^b^ | 0,0009245  (0,0001750–0,0034289) ^c^ | 0,0017972  (0,0011661–0,0045434) ^d^ | **0.03** |
| *ALOX15* | 0,0035394 ± 0,0056283 | 0,0043005 ± 0,0054841 | 0,005861 ± 0,0085727 | 0,0039332 ± 0,0055004 | 0.52 |
| *BCL2A1* | 0,0349814 ± 0,0215721 | 0,0341094 ± 0,0206643 | 0,0467224 ± 0,0374928 | 0,0563449 ± 0,0481085 | 0.04 |
| *BCL2L1* | 0,0469843 ± 0,0644859 | 0,0503294 ± 0,0716647 | 0,0564726 ± 0,0723487 | 0,03837 ± 0,0442316 | 0.78 |
| *CA1* | 0,101212 ± 0,1369527 | 0,0937127 ± 0,1452878 | 0,1130281 ± 0,2120752 | 0,1078491 ± 0,1421796 | 0.96 |
| *IRS2* | 0,0080088 ± 0,0055126 | 0,0085255 ± 0,0058547 | 0,0081185 ± 0,0058616 | 0,0079568 ± 0,0060999 | 0.98 |
| *MMP9* | 0,0151766 ± 0,010369 | 0,018591 ± 0,0122833 | 0,0177008 ± 0,0149315 | 0,0182883 ± 0,0107291 | 0.67 |
| *ECHDC3* | 0,000529 ± 0,0004019 | 0,0007 ± 0,0005325 ^e^ | 0,0005899 ± 0,0005088 | 0,0003699 ± 0,0002482 ^f^ | 0.08 |
| *MYL4* | 0,0646878 ± 0,1009217 | 0,0718188 ± 0,1311822 | 0,0602537 ± 0,0728169 | 0,0463882 ± 0,0446666 | 0.81 |
| *IL18R1* | 0,0003268 ± 0,0001745 | 0,0003645 ± 0,0002113 | 0,0003526 ± 0,0001323 | 0,0003577 ± 0,000213 | 0.86 |
| *AREG* | 0,0007965 ± 0,0015157 | 0,0006161 ± 0,0009986 | 0,0007569 ± 0,0012826 | 0,0007929 ± 0,0010474 | 0.95 |

**Supplementary Table 1.** Gene expression classified according to coronary lesion extension.

Data are presented as the mean ± standard deviation or median (range). Parametric analysis was conducted by ANOVA. For non-parametric testing, the Kruskal–Wallis test was used. Categorical variables were compared using the Chi-square test. ‘Without’, Friesinger index 0; ‘Low’, Friesinger index 1–5; ‘Intermediate’, Friesinger index 6–10; ‘Major’, Friesinger index 11–15.

^ad^***p* = 0.006**, without vs major extension of coronary lesion by Mann–Whitney test.

^bd^***p* = 0.03**, low vs major extension of coronary lesion by Mann–Whitney test.

^cd^***p* = 0.01**, intermediate vs major extension of coronary lesion by Mann–Whitney test.

^ef^***p =* 0.05**, low vs major extension of coronary lesion by post-hoc Tukey test.

**Supplementary Table 2.** Clinical and laboratory data of patients classified according to *TREML4* mRNA expression.

|  | ***TREML4 mRNA* expression** | |  |
| --- | --- | --- | --- |
| **Variables** | **Low** | **High** | ***p*** |
| Age, years | 59 ± 9 | 59 ± 11 | *0.972* |
| Sex male, % | 65.5 (19) | 59.3 (16) | *0.063* |
| BMI, kg/m^2^ | 27.7 ± 3.4 | 29.0 ± 6.5 | *0.346* |
| Obesity, % | 24.1 (7) | 25.9 (7) | *0.946* |
| Dyslipidemia, % | 85.2 (23) | 92.9 (26) | *0.362* |
| Diabetes, % | 20.7 (6) | 51.9 (14) | ***0.015*** |
| Hypertension, % | 82.8 (24) | 81.5 (22) | *0.901* |
| Diastolic pressure, mmHg | 85 ± 11 | 86 ± 20 | *0.453* |
| Systolic pressure, mmHg | 141 ± 21 | 149 ± 24 | *0.222* |
| Physical activity, % | 41.4 (12) | 37.0 (10) | *0.740* |
| Alcohol intake, % | 34.5 (10) | 19.2 (5) | *0.205* |
| Smoking, % | 31.0 (9) | 14.8 (4) | *0.151* |
| Family history of CAD, % | 41.4 (12) | 51.9 (14) | *0.432* |
| Glucose, mg/dL | 99.8 ± 25.5 | 129.6 ± 64.4 | *0.076* |
| Total cholesterol, mg/dL | 191.5 ± 59.1 | 166.3 ± 55.1 | *0.105* |
| HDL cholesterol, mg/dL | 37.1 ± 14.3 | 32.4 ± 10.3 | *0.120* |
| LDL cholesterol, mg/dL | 121.6 ± 48.3 | 99.4 ± 50.9 | *0.104* |
| Triglycerides, mg/dL | 173.6 ± 97.7 | 172.3 ± 72.8 | *0.646* |
| AST, U/L | 30.9 ± 16.7 | 27,9 ± 10.8 | *0.717* |
| ALT, U/L | 31.4 ± 23.3 | 30.9 ± 17.9 | *0.941* |
| Urea, mg/dL | 38.8 ± 11.3 | 37.8 ± 14.1 | *0.775* |
| Creatinine, mg/dL | 0.96 ± 0.33 | 0.87 ± 0.3 | *0.281* |
| Uric acid, mg/dL | 5.1 ± 1.6 | 4.3 ± 1.5 | 0.086 |
| Antihypertensive, % | 69.0 (20) | 66.7 (18) | *0.854* |
| Anticoagulants, % | 31.0 (9) | 22.2 (6) | *0.457* |
| Antidiabetics, % | 6.9 (2) | 51.9 (14) | ***0.001*** |
| Statins, % | 25.0 (7) | 32.1 (9) | *0.554* |

Data are shown as the mean ± standard deviation or the percentage for categorical variables (number of patients). Parametric analysis was performed using the t-test. For non-parametric testing, the Mann–Whitney test was used. Categorical variables were compared by the Chi-square test. BMI, body mass index; HDL, cholesterol high-density lipoprotein; LDL, cholesterol low density lipoprotein; AST, aspartate aminotransferase; ALT, alanine transaminase. ‘Low’, *TREML4* mRNA expression below the median; ‘high’, *TREML4* mRNA expression above the median.
